# Ubiquitin Carboxyl-Terminal Hydrolases (UCHs): Potential Mediators for Cancer and Neurodegeneration

**DOI:** 10.3390/ijms21113910

**Published:** 2020-05-30

**Authors:** Amit Sharma, Hongde Liu, Fabian Tobar-Tosse, Tikam Chand Dakal, Michael Ludwig, Frank G. Holz, Karin U. Loeffler, Ullrich Wüllner, Martina C. Herwig-Carl

**Affiliations:** 1 Department of Ophthalmology, University Hospital Bonn, 53127 Bonn, Germany; Amit.Sharma@ukbonn.de (A.S.); Frank.Holz@ukbonn.de (F.G.H.); Karinloeffler@uni-bonn.de (K.U.L.); 2 State Key Laboratory of Bioelectronics, School of Biological Science & Medical Engineering, Southeast University, Nanjing 210096, China; liuhongde@seu.edu.cn; 3 Department of Basic Health Sciences, Pontificia Universidad Javeriana Cali, 760031 Cali, Colombia.; ftobar@javerianacali.edu.co; 4 Department of Biotechnology, Mohanlal Sukhadia University, Rajasthan 313001, India; tikam260707@gmail.com; 5 Department of Clinical Chemistry and Clinical Pharmacology, University Hospital of Bonn, 53127 Bonn, Germany; mludwig@uni-bonn.de; 6 Department of Neurology, University Hospital Bonn, 53127 Bonn, Germany; ullrich.wuellner@ukbonn.de; 7 German Center for Neurodegenerative Diseases (DZNE), 53127 Bonn, Germany

**Keywords:** cancer, neurodegenerative diseases, ubiquitin carboxyl-terminal hydrolases (UCHs), Parkinson’s disease, BAP1, alpha-synuclein

## Abstract

Emerging evidence suggests an inverse association between cancer and neurodegenerative diseases (NDD). Although phenotypically different, both diseases display a significant imbalance in the ubiquitination/deubiquitination processes. Therefore, we particularly investigated the expression of ubiquitin C-terminal hydrolases (UCHs*: UCH-L1, UCH-L3, UCH-L5* and *BAP1*)*,* a subfamily of deubiquitinating enzymes (DUBs), using publically available datasets (GTEx, TCGA) and observed altered expression of *UCH*-*L1*, *UCH-L3, UCH-L5* in 17 cancer types. Interestingly, *UCH-L1* (known to be enriched in neurons and interacting with the Parkinson’s disease-associated protein α-synuclein) appeared to be a prognostic indicator of unfavorable outcome in endometrial and urothelial cancer, while increased expression of *UCH-L3* and *UCH-L5* was associated with poor survival in liver and thyroid cancer, respectively. In normal tissues, *UCH-L1* was found to be strongly expressed in the cerebral cortex and hypothalamus, while *UCH-L3* expression was somewhat higher in the testis. The occurrence of mutation rates in UCHs also suggests that *BAP1* and *UCH-L5* may play a more dominant role in cancers than *UCH-L1* and *UCH-L3*. We also characterized the functional context and configuration of the repeat elements in the promoter of DUBs genes and found that UCHs are highly discriminatory for catabolic function and are mainly enriched with LINE/CR1 repeats. Regarding the thesis of an inverse association between cancer and NDD, we observed that among all DUBs, UCHs are the one most involved in both entities. Considering a putative therapeutic potential based on presumed common mechanisms, it will be useful to determine whether other DUBs can compensate for the loss of UCH activity under physiological conditions. However, experimental evidence is required to substantiate this argument.

## 1. Background

Ubiquitination, the covalent attachment of ubiquitin (ub) to protein substrates, is achieved by the sequential action of three enzymes: E1 ubiquitin-activating enzyme; E2 ubiquitin-conjugating enzyme; and E3 ubiquitin ligase. This attachment can occur at one or more lysine sites, resulting in monoubiquitination, which can remain as it is or be further extended by additional ubiquitin molecules to form elongated chains (polyubiquitination). This ultimately leads to the degradation of unwanted proteins, altered subcellular localization/function and protein interactions. Deubiquitinating enzymes (DUBs) can efficiently remove ubiquitin from the protein and thus help to maintain a dynamic balance between ubiquitination and deubiquitination within the cellular environment which is essential for the efficient maintenance of cellular functions.

DUBs play several other functional roles besides deubiquitination, especially in cancer [1]. For instance, among the UCHs or ubiquitin carboxy-terminal hydrolases, a subfamily member of DUBs, *BAP1* (*BRCA1-associated protein 1*) possesses unique ability as a tumor suppressor, while others (*UCH-L1, UCH-L3, UCH-L5*) are characterized as tumor promoters [2,3,4,5,6]. Notably, almost three decades ago, UCH-L1 was found to be abundant in neurons and has been linked to neurodegenerative diseases [7]. Several studies have also pointed to the peculiar role of other members of DUBs in neurodegeneration (e.g., Machado–Joseph disease (MJD)/Spinocerebellar ataxia type 3 (SCA3)) [8,9,10]. In a broader perspective, epidemiological studies have also discussed the diverse aspects of neurodegeneration and cancer, primarily, to underline the lower incidence of neurodegenerative disorders (NDDs) in cancer survivors and vice versa [11]. Though the clinical presentations of these diseases are strictly different, a significant overlap has been observed between the genes upregulated in CNS disorders and downregulated in cancers, and vice versa [12]. Nevertheless, the priority aspect for the healthy cells (timely division to avoid increased risk of cancer) and the aging neurons (to achieve longevity without further divisions) is compromised in these two devastating conditions.

Since most studies published thus far are from mouse and cell lines, herein, we put emphasis on the cancer-specific characteristics of UCHs by using publically available databases of healthy humans (GTEx) , cancer patients (TCGA) and NDDs. With regard to cancer and neurodegeneration, this is the first study to show that UCHs may contribute significantly to cancer. The highly discriminatory catabolic function of UCHs and the enrichment of LINE/CR1 (CR1 subfamily of LINE elements) repeats have not been reported before.

## 2. Results and Discussion

**Figure 1:**
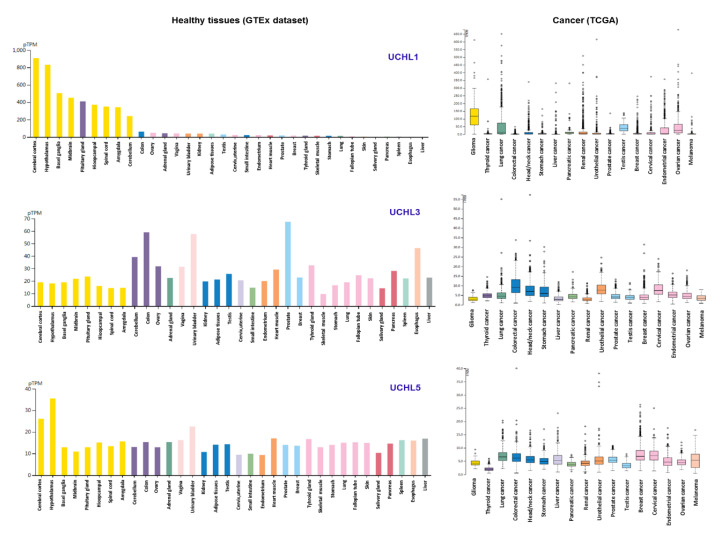
The expression analysis of ubiquitin C-terminal hydrolases (UCHs) *UCH-L1*, *UCH-L3*, and *UCH-L5* in healthy tissues and in 17 cancer types. The expression analysis of *UCH-L1*, *UCH-L3*, *UCH-L5* in 34 healthy tissues extracted from GTEx database. The expression is represented with TPM (tags per million clean tags). The clear enrichment of *UCH-L1* in CNS and equal distribution of *UCH-L3*, *UCH-L5* in all tissue types is shown (Left panel). The expression analysis of *UCH-L1*, *UCH-L3*, *UCH-L5* in 17 cancer types extracted from The Cancer Genome Atlas (TCGA) project (https://portal.gdc.cancer.gov/). Boxplot indicates gene expression fragments per kilobase of exon per million reads (FPKM) in cancer samples (Right panel).

*UCH-L1* (also known as *PARK5* and *PGP9.5*) was previously found to be enriched in neurons, and was shown to promote alpha-synuclein neurotoxicity in Parkinson’s disease (PD) patients [13]. Primarily, the association of *UCH-L1* and PD was mainly supported by the I93M mutation in a German family [14,15]. The second mutation (*UCH-L1**^GLU7ALA^***) was found in a Turkish family, mainly related to the occurrence of a neurodegenerative syndrome in childhood, not exclusively PD [15]. It has also been suggested that the polymorphism in the *UCH-L1* gene (S18Y) may play a protective role in Parkinson’s disease, but recent studies have shown contradictory results [16]. Therefore, the altered function of UCH-L1 on a broad scale cannot be regarded as being limited to PD, but as a general consequence of neurodegeneration. This is supported by data in Alzheimer’s disease (AD), where UCH-L1 was also found to be interacting with amyloid β precursor protein (APP) [17]. Furthermore, there are also reports about *UCH-L1* expression in the neuroretina and in lens epithelial cells of atopic cataracts [18,19]. It is noteworthy to mention that *UCH-L1* has also been associated with cancer and therefore was proposed as an oncogene [20,21]. Considering this dilemma regarding the exact contribution of UCH-L1, we have verified *UCH-L1* expression in 35 tissues of healthy individuals using the publicly available GTEx database. Our results show that a high expression of *UCH-L1* is related to CNS-related tissues, which is consistent with previous studies (Figure 1, Left panel). Importantly, we found a variable expression rate of *UCH-L1* in the tissues of 17 cancer types (Figure 1, Right panel). Further analysis revealed that in terms of patient survival, *UCH-L1* appears to be an unfavorable indicator for endometrial cancer (*p =* 0.000041*)* and urothelial cancer *(p =* 0.00019*)*. Hence, it may be concluded that the altered expression of *UCH-L1* is a disease-related consequence, particularly in cancer. It is noteworthy to mention that we cannot determine if the mutations/loss of *UCH-L1*, which is associated with cancer, has an additional effect on neurodegenerative diseases (UCHs alone or in combination with the major dysregulated proteins such as α synuclein, APPs, etc.) or vice versa.

**Figure 2:**
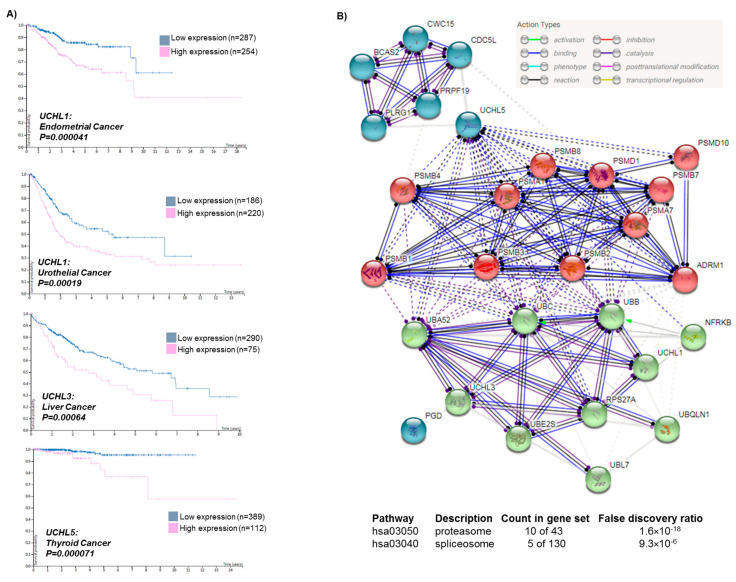
Survival analysis for UCHs in cancer types (for TCGA) and pathway enrichment analysis. (**A**) *UCH-L1* as prognostic indicator for the unfavorable outcome in the endometrial and urothelial cancer, while *UCH-L3* and *UCH-L5* is associated with poor survival in liver and thyroid cancer. Survival difference analysis was done with log-rank test in a Kaplan–Meier survival model. (**B**) Pathway enrichment of UCHs and their functional partners constructed by the String tool is shown. Only the experimentally tested interaction was used for cluster formation, and two main pathways involved in the proteasome and spliceosome are also shown (KEGG ID: hsa03050 and hsa03040).

Unlike *UCH-L1*, the expression of the other two members, *UCH-L3* and *UCH-L5* (also called *UCH37*), was found not to be restricted to CNS, but was also observed in other anatomical regions (Figure 1, Left panel). Previously, it was shown that *UCH-L3* is altered in cell lines of multiple malignancies such as breast cancer, prostate cancer, and ovarian cancers, while *UCH-L5* has been implicated in epithelial ovarian cancer and hepatocellular carcinoma [3]. We found additional cancer types with altered expression of *UCH-L3* and *UCH-L5* (Figure 1, Right panel). A high expression of *UCH-L3* and *UCH-L5* was an indicator of poor survival for liver (*p* = 0.00064) and thyroid cancer (*p* = 0.000071) (Figure 2A). However, only *UBA52* lacks prognostic ability, while a high expression of *UBL7* and *UBB* proved to be favorable for survival compared to others (Table S1).

We further identified the functional partners of UCH-L1/3/5 and constructed an interaction network using the String tool (top, Figure 2B). The analysis revealed that the UCHs are tightly linked and involved in known proteasome- and spliceosome-related pathways (*p* = 1.6 × 10^−18^ and 9.3 × 10^−6^, respectively). In particular, UCH-L5 was involved in the splicing pathway by binding to the proteins PLRG1 and CDC5L, which are the components of the cell division cycle 5-like complex and are required for pre-mRNA splicing. Meanwhile, UCH-L1 and UCH-L3 were found to be mainly involved in the proteasome pathway through highly associated ubiquitins, such as UBA52, UBB, UBC, RPS27A (UBA80), and UBE2S (below, Figure 2B), which target cellular proteins for degradation by the 26S proteasome. UCH-L1, UCH-L3, and UCH-L5, however, are also closely associated with subunits (PSMD1, PSMB8, PSMB2, PSMA1, etc.) of the ring-shaped 20S core structure of the proteasome (middle, Figure 2B). Taken together, these three UCHs are involved in the process of splicing and protein degradation, including the processing of immunoproteasomes.

With regard to *BAP1*, we have recently shown gene expression and survival prediction in several other cancers types (*n* = 29) in addition to uveal (eye) melanoma [6]. Herein, we further investigated the incidence of UCH mutation rate in the cancer genome and observed that *BAP1* (2032 mutations) and *UCH-L5* (1276 mutations) harbor many more mutations than *UCH-L3* (556 mutations) and *UCH-L1* (456 mutations), respectively. In summary, DUBs, especially UCHs, seem to be nonspecifically altered in different types of cancer, while they show more specific alterations in NDDs (PD, AD, SCA3). To further verify this, we accessed the differential expression of DUBs in NDDs (Supplementary Figure S1). As expected, two DUBs (*USP36* and *USP21*) were found to be altered in PD, while only a few also showed variable expression in AD samples compared to healthy controls. However, the changes in NDDs were not as noticeable as in cancer.

**Figure 3:**
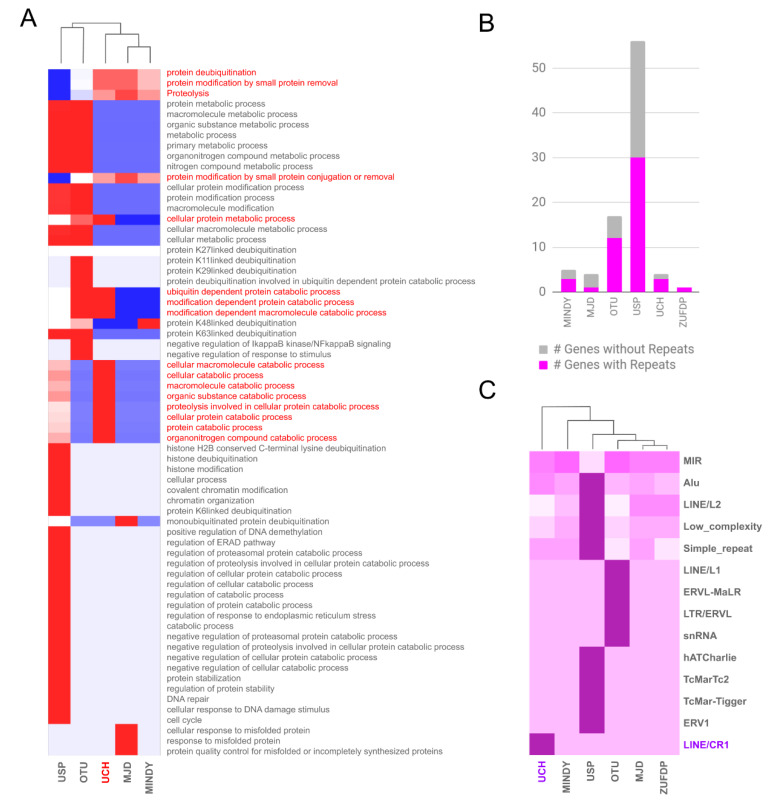
Functional and structural analysis of the deubiquitinating enzymes (DUBs) genes. (**A**) Gene ontological categories associated with each subfamily of DUBs, a hierarchical tree connects the subfamilies and the heat map shows the highest (red) and lowest (blue) associated categories. (**B**) Absolute frequency of DUB subfamilies with repeats in the upstream promoter region. (**C**) Heatmap of correlation between DUB subfamilies and types of repeats (dark purple shows high association).

The functional analysis of the DUBs family using gene ontology reveals two main associations: protein or histone deubiquitination as a ubiquitous function for all DUBs (with specific targets for each) and metabolic processes for specific subfamilies such as the UCHs (Figure 3A). Although UCH is associated with MINDY and MJD based on the functional categories, the high number of catabolic functions makes it strictly different from the others. To determine the possible reason for this functional deviation in UCHs, we investigated the upstream promoter region of the DUBs family by examining the DNA repeats. The analysis of the subfamilies revealed that USP with 46% genes and OTU with 29% genes had no repeat sequence in their promoter. In the case of UCHs, only the *UCH-L5* gene lacks repeats; similarly, in the MJD, only the *JOSD2* gene showed the same pattern. The limited pattern of repeats was also visible in one unique gene, *ZUFDP* (Figure 3B). We have further defined the categories of these repeat types and found that USP contains the most divergent repeat family, especially Alu and LINEs, while UCHs exclusively showed an association with rare LINE/CR1 types of ancient repeats (Figure 3C). Further investigations also revealed that *UCH-L3* (in intron 1,5,6) and *UCH-L1* (in upstream promoter region) contain a significantly high number of LINE/CR1 (Supplementary Figure S2). In contrast, *BAP1* harbors only one LINE/CR1 in the upstream promoter region, and *UCH-L5* lacks these elements. The unique LINE/CR1 of the *BAP1* gene was found to be one of the most ancestral LINEs. We also found a high density of transcription factor binding sites (133 in total) in the structure of these LINEs, with 28 being the most significant. Among the regulatory elements Cdx-1, C/EBPalpha, Ncx, and C/EBPdelta were found to highly distinctive to all LINE/CR1, especially the UCH-related LINE/CR1 was found to be associated with embryonic development and morphogenesis.

It is well known that the families and subfamilies of LINE elements, which are abundant in the genome and often found in the vicinity of prime genes associated with human diseases, are also associated with cancer [22,23]. The CR1 subfamily of LINE elements was previously reported to be significantly upregulated in monocytes and B cells from systemic lupus erythematosus (SLE) patients [24]. From the current study, we do not know if LINE/CR1 affects the activity or the expression (tissue specific or locus specific) of UCHs.

## 3. Conclusions

Among all DUBs, UCHs are the ones most involved in cancer and NDD. Importantly, the contribution of highly discriminatory catabolic function of UCHs and the enrichment of LINE/CR1 repeats needs further investigation. In a broader prospective, it will be useful to determine whether other DUBs can compensate the loss of UCH activity under physiological conditions. The findings we have presented here require experimental evidence to pave the way for a deeper understanding of the mechanisms connecting cancer and neurodegeneration.

## 4. Material and Methods

The expression analysis of *UCH-L1*, *UCH-L3*, *UCH-L5* in 34 healthy tissues was extracted from the GTEx database. Briefly, the RNA-seq tissue data generated by the Genotype-Tissue Expression (GTEx) project are reported as mean pTPM (protein-coding transcripts per million), hence corresponding to the mean values of the different individual samples from each tissue. String analysis was performed using online server (https://string-db.org/). First, UCHs (UCH-L1/3/5) were specified and the functional partners of UCH-L1, UCH-L3, and UCH-L5 were identified using the online server. Only the experimentally-validated interactions were selected to establish the network. In the first level, only the ten most important interaction partners were retained, while five partners were kept for the second level. Then, the network was clustered with the k-means method and enrichment was further carried out for these selective proteins and their functional partners using STRING function.

Gene expression datasets to evaluate DUBs in NDDs (AD: GSE13214, Case/Controls = 13/10; hippocampus and cortex frontal; PD: GSE24378, Case/Controls = 8/9, Dopamine neurons) were retrieved from GEO (Gene Expression Omnibus) (http://www.ncbi.nlm.nih.gov/geo/). The *p*-value was called with two-sample *t*-test between normal samples and disease samples and the plot of *p*-value (-log10) against fold change (log2) was drawn.

For cancers, The Cancer Genome Atlas (TCGA) data was accessed. Briefly, the RNA-seq data in 17 cancer types reported as median FPKM (number Fragments per Kilobase of exon per Million reads), generated by TCGA. In addition, the comprehensive cancer mutation database COSMIC (http://cancer.sanger.ac.uk) was used to check the mutational rate among UCHs.

In order to perform a functional and structural analysis of the DUB genes, the list of genes by subfamily was retrieved from HUGO (https://www.genenames.org/). The functional analysis was performed using gene ontology categories, all GO levels were included and normalized by using hypergeometric distribution for subfamilies. For repeats in the promoter of the DUB genes, the position of genes and repeats annotated in the Human Genome Reference (GRCh38.p7) were used. Hierarchical clustering was applied by using simple linkage and Pearson correlation methods. The standard maximum parsimony method was used to grade conservation and homology among these UCH-related LINEs for the phylogenetic tree. The possible regulatory sites in the structure of these LINEs were identified by using the PROMO dataset, using the link http://alggen.lsi.upc.es/.

## Supplementary Materials

The following are available online at https://www.mdpi.com/1422-0067/21/11/3910/s1, Figure S1: Gene expression of DUBs in neurogenerative diseases. Gene expression data showing an alteration of DUBs in Alzheimer’s (right) and Parkinson’s Diseases (Left). The *p*-value was calculated with two-sample *t*-test between normal samples and disease samples and the plot of *p*-value (−log10) against Fold change (log2) is drawn. Figure S2: LINE/CR1 and regulatory sites in UCHs. A. The density and distribution of LINE/CR1. B. LINE/CR1 as a common element in UCH family in comparison with other UCH-related LINEs. C. Contrary to L1 and L2 LINEs, LINE/CR1 shows heterogeneity and distribution in several branches. D. Transcription factors and the LINE/CR1 in each UCH gene. Table S1: The prognostic ability for selective UCH genes.

## Abbreviations

USPs	ubiquitin-specific proteases
OTU	ovarian tumor proteases
MJDs	Machado–Josephin domain proteases
MINDYs	(motif interacting with Ub-containing novel DUB family)
ZUFDP	Zn-finger and UFSP domain protein
UCHs	ubiquitin C-terminal hydrolases
JAMM	Jab1/Mov34/Mpr1 Pad1 N-terminal+ (MPN+)
SCA3	Spinocerebellar ataxia type 3
