# Supplementary material for: Ubiquitin Carboxyl-Terminal Hydrolases (UCHs): Potential Mediators for Cancer and Neurodegeneration
Source: Int J Mol Sci. 2020 May 30;21(11):3910. doi: 10.3390/ijms21113910 (PMC7312489; doi:10.3390/ijms21113910)
Supplement: Supplementary file 1 [file ijms-21-03910-s001.zip › Supplementary table 1.docx]

**Table S1**

| **Genes** | **Cancer types** | **High expression & prognosis** |
| --- | --- | --- |
| UBB | Renal cancer | Favorable |
| UBC | Renal cancer | Unfavorable |
| UBA52 | / | / |
| PSMB4 | Renal cancer, Liver cancer | Unfavorable |
| PSMD1 | Liver cancer, Lung cancer | Unfavorable |
| PSMA7 | Liver cancer | Unfavorable |
| UBE2S | Renal, Liver & Endometrial cancer | Unfavorable |
| CDC5L | Melanoma | Unfavorable |
| UBL7 | Cervical cancer | Favorable |
