# Supplementary figures and images for: Ubiquitin Carboxyl-Terminal Hydrolases (UCHs): Potential Mediators for Cancer and Neurodegeneration
Source: Int J Mol Sci. 2020 May 30;21(11):3910. doi: 10.3390/ijms21113910 (PMC7312489; doi:10.3390/ijms21113910)

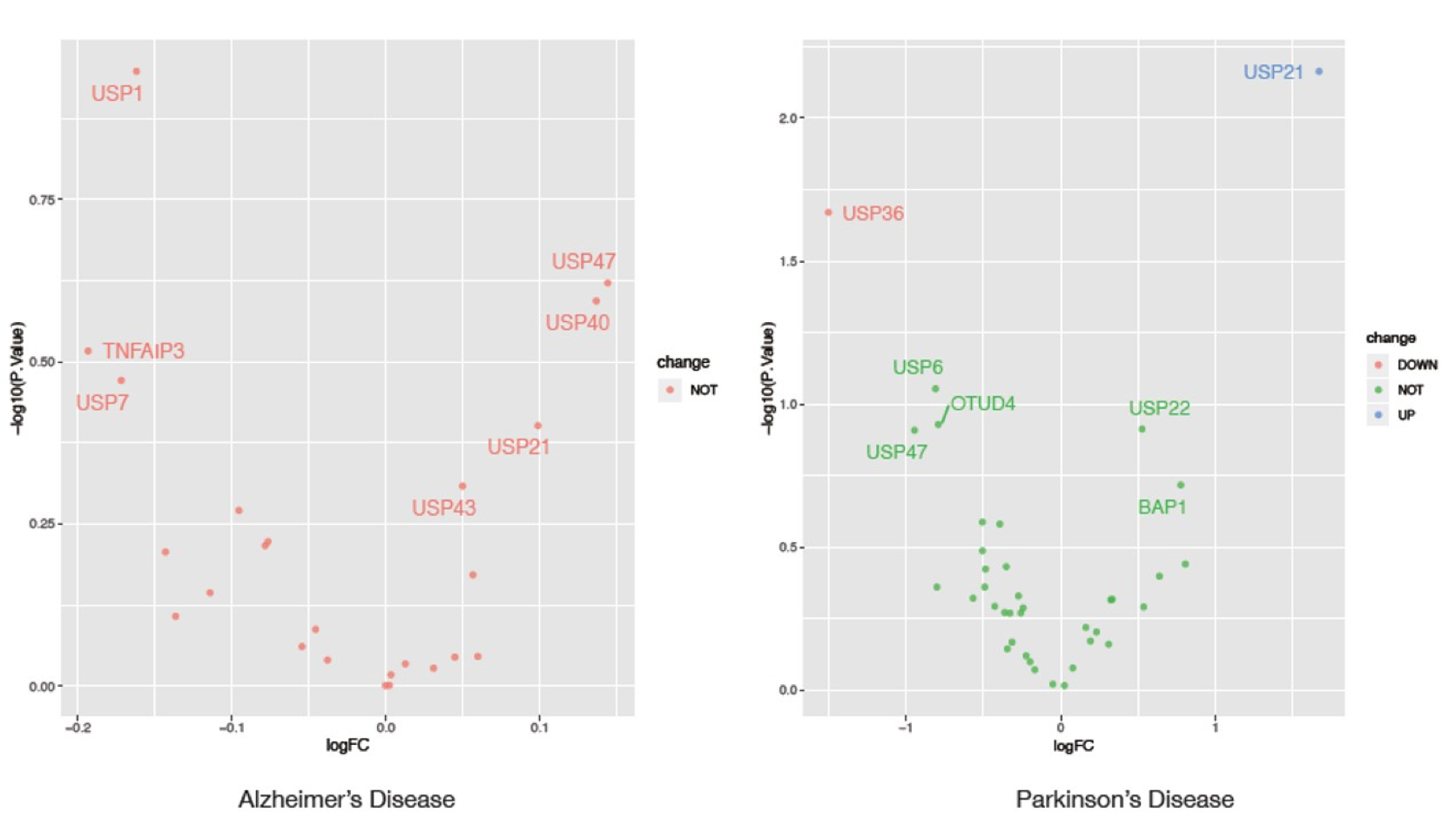

Supplement: Supplementary file 1 [file ijms-21-03910-s001.zip › Supplementary figure 1.png]

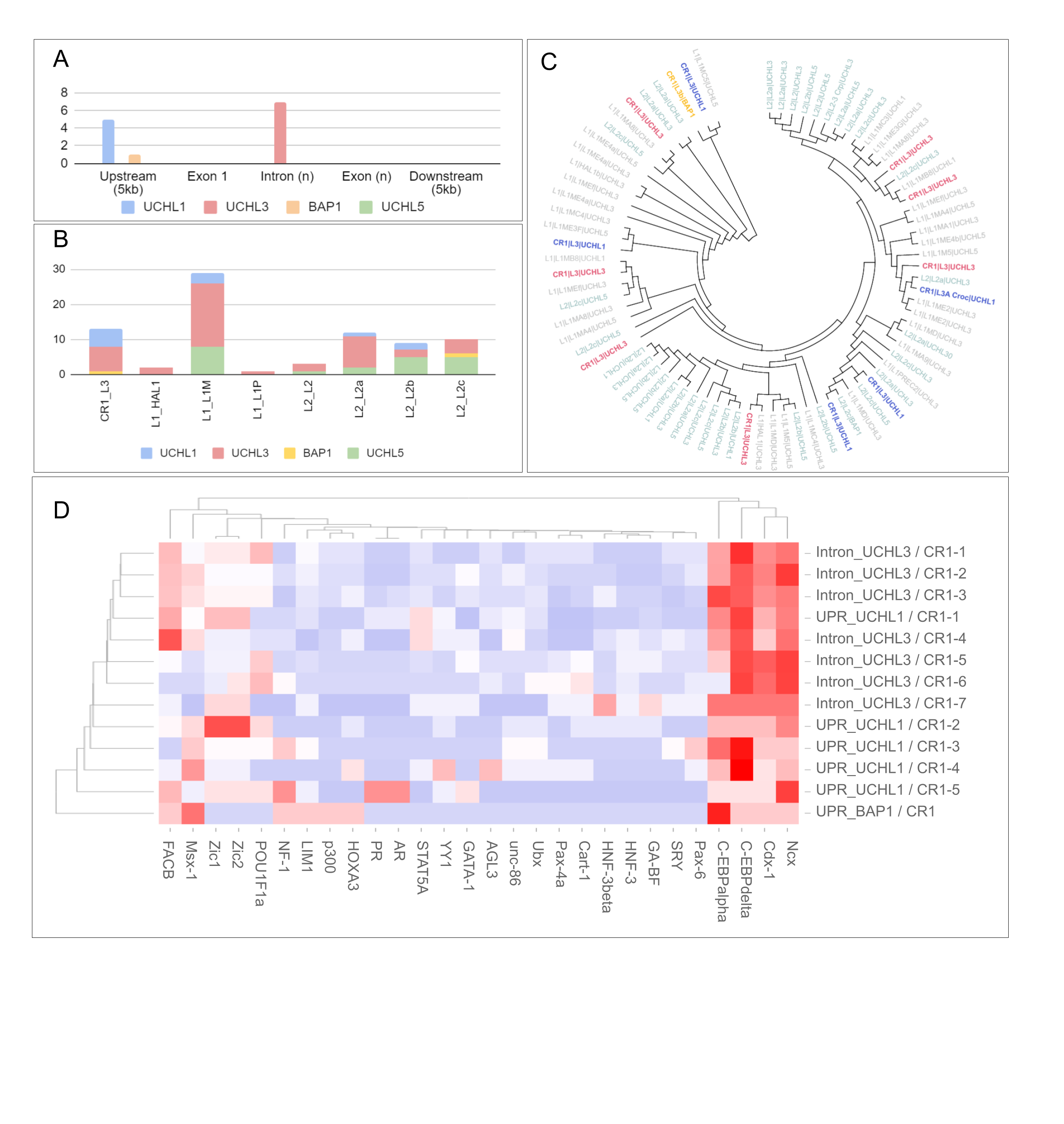

Supplement: Supplementary file 1 [file ijms-21-03910-s001.zip › Supplementary figure 2.png]
